# Molecular characterization and antibiotic resistance profiles of *Salmonella* isolated from fecal matter of domestic animals and animal products in Nairobi

**DOI:** 10.1186/s40794-016-0045-6

**Published:** 2017-01-13

**Authors:** Diana Nyabundi, Nyamongo Onkoba, Rinter Kimathi, Atunga Nyachieo, Gerald Juma, Peter Kinyanjui, Joseph Kamau

**Affiliations:** 1 Department of Biochemistry, School of Medicine, University of Nairobi, P.O. Box 30197–00100, Nairobi, Kenya; 2 Institute of Primate Research, P.O. Box 24481–00502, Karen, Nairobi, Kenya

**Keywords:** *Salmonella*, Domestic animals, Phylogeny, Resistance

## Abstract

**Background:**

*Salmonella* has significant public health implications causing food borne and zoonotic diseases in humans. Treatment of infections due to *Salmonella* is becoming difficult due to emergence of drug resistant strains. There is therefore need to characterize the circulating non-typhoidal *Salmonella* (NTS) serovars in domestic animals and animal products in Kenya as well as determine their antibiotic resistance profiles.

**Methods:**

A total of 740 fecal samples were collected from cows (*n* = 150), pigs (*n* = 182), chicken (*n* = 191) and chicken eggs (*n* = 217) from various markets and abattoirs in Nairobi. The prevalence of NTS serovars using culture techniques and biochemical tests, antimicrobial sensitivity testing using disc diffusion method of the commonly prescribed antibiotics and phylogenetic relationships using 16S rRNA were determined.

**Results:**

The results showed that the overall prevalence of *Salmonella* was 3.8, 3.6, 5.9 and 2.6% for pigs, chicken, eggs and cows respectively. Two serovars were isolated *S.* Typhimurium (85%) and *S.* Enteritidis (15%) and these two serovars formed distinct clades on the phylogenetic tree. Forty percent of the isolates were resistant to one or more antibiotics.

**Conclusion:**

The isolation of *Salmonella* Typhimurium and *Salmonella* Enteritidis that are resistant to commonly used antibiotics from seemingly healthy animals and animal products poses a significant public health threat. This points to the need for regular surveillance to be carried out and the chain of transmission should be viewed to ascertain sources of contamination.

## Background

*Salmonella* is a gram-negative enteric bacteria and is one of the major zoonotic foodborne pathogens worldwide [1]. Non typhoidal Salmonella (NTS) is responsible for about 93.8 million cases and causes approximately 155,000 deaths [2] as well as economic losses in the agricultural sector [3]. Non typhoidal serovars comprise of host generalists serovars such as *S.* Typhimurium and *S*. Enteritidis that induce a self-limiting gastroenteritis in a broad range of unrelated host species or can be adapted to a particular host such as *S.* Gallinarium in poultry and *S.* Dublin in cattle [4]. Non typhoidal salmonellosis has been associated with consumption of contaminated foods of animal origin, such as poultry, swine, dairy products [5] as well as person to person contact [6]. In developed countries, NTS in humans generally causes self-limiting gastroenteritis, in sub-Saharan Africa however NTS causes an invasive kind of infection that is common in infants, young children and adults. Salmonellosis also shares co-morbidities with tropical infectious diseases like malaria and Human Immunodeficiency Virus [7]. The invasive type is mainly caused by a variant ST313 that occurs exclusively in sub Saharan Africa [8]. The situation is further being complicated by emergence of drug resistant strains compromising the clinical treatment of the disease [9]. Drug resistance occurs as a result of the unmonitored use of antibiotics in farms for prophylaxis or as growth promoters (feed additives) [10, 11]. Multi drug resistance to commonly available drugs used as first line drugs which include chloramphenicol, trimethoprim/sulphamethoxazole and selected beta lactamases as well as fluoroquinolones is widespread in Kenya and Malawi [7]. The resistance observed among the invasive strains is conferred largely through a plasmid [11].

In Kenya, the prevalence of NTS serovars circulating in farms and animal products is unknown. [12]. Of the few studies done in Kenya, none has determined prevalence of Salmonella in eggs. The present study therefore sought to determine prevalence of Salmonella serovars circulating in cows, pigs, chicken and eggs across Kenya using molecular tools as well as determine the antibiotic resistant profiles of commonly available drugs to the *Salmonella* isolates namely; tetracycline, nitrofurantoin, nalidixic acid, streptomycin, sulphamethoxazole, cotrimaxazole, gentamycin and ampicillin.

## Methods

Approximately 5 g of fresh fecal samples from cows (*n* = 150) and pigs (*n* = 182), chicken cloacal swabs (*n* = 191) and eggs (*n* = 217) were collected for this study from December 2013 to October 2014. Five grams of faeces from the cows and pigs were collected aseptically from the rectum immediately after the animals were slaughtered. Cloacal swabs were collected asceptically from chicken both in the slaughter houses and in the local markets in Nairobi County. The eggs were collected from the same markets that the chicken sampling was carried out and put in a sterile jar and transported to the lab for further processing.

Bovine fecal matter was collected at Nairobi’s Dagoretti slaughter house complex. Cattle slaughtered here originated from different parts of the country [13]. The pigs slaughtered at Nairobi’s Ndumbuini abattoir originate from Nairobi and Kiambu counties which are among the main pig farming counties in Kenya [14]. Grade and indigenous chicken samples as well as eggs were collected from various markets and slaughterhouses in Nairobi and its environs that receive chicken and eggs from all parts of the country. The sampling was done according to Daniel et al. [15]:

$$ \boldsymbol{n}=\frac{{\boldsymbol{Z}}^2\boldsymbol{P}\left(1-\boldsymbol{P}\right)}{{\boldsymbol{d}}^2} $$

Where *n* = sample size, Z = Z statistic for a level of confidence, *P* = expected prevalence or proportion, d = precision

Using this formula the sample sizes was obtained: Pigs = 182 [16], Chicken- 191 [17], Eggs = 217 [18], Cattle = 148 [19].

### Isolation and identification of *Salmonella*

In the laboratory, 1 g of the fecal matter from cows and pigs as well as cloacal swabs of chicken was inoculated in 10 ml of selenite broth (Oxoid, UK) and incubated at 37 °C for 24 h. After 24 h, a loopful of the enriched sample was plated on XLD agar plates (Oxoid, UK) and incubated at 37 °C for 24–48 h.

The eggs shells were cleaned thoroughly with soap and wiped with 70% ethanol. After air drying them, the eggs were cracked open using a pen knife and its contents thoroughly mixed after which 1 ml of the contents was cultured in 25 ml buffered peptone water (International Diagnostic Group, Lancashire, UK) and incubated at 37 °C for 24 h. An aliquot of 1 ml of the pre enriched sample was re-cultured in 10 ml of selenite F broth incubated 37 ° C for 24 h. After 24 h of incubation, a loopful of the selenite F broth culture was streaked on XLD agar plates and incubated at 37 °C for 24 to 48 h. The XLD plates were examined for the presence of *Salmonella* colonies. Positive samples were subsequently to biochemical tests using the API Biomerieux 20E strips, (Marcy-l’Etoile, France).

### Extraction of genomic DNA

Pure colonies of Salmonella cultured in nutrient broth (Oxoid, UK) was used for DNA extraction using the QIAprep miniprep kit (Qiagen Valencia CA, USA) according to the manufacturers’ instructions.

### Polymerase chain reaction (PCR)

Forward primer 16SF1 (5′-TGTTGTGGTTAATAACCGCA-3′) and reverse primer; 16SIII (5′-CACAAATCCATCTCTGGA-3′) of the 16S rRNA gene (Inqaba Biotech, South Africa) were used to amplify the 572 bp PCR product. Amplifications were carried in 50 μl reaction volumes containing; 25 μl of Dream Taq Master Mix (Thermoscientific, USA), 15 μl of nuclease free water, 2.5 μl of each primer and 2.5 μl of the extracted bacterial DNA. The amplifications were done in 35 cycles with an initial denaturation at 95 °C for 5 min, a denaturation step of 95 °C for 2 min, primer annealing at 55 °C for 30 s and primer extension at 72 ° C for 1 min. Finally, an additional extension was done for 10 min at 72 °C. The PCR products were visualized on 2% agarose gel with ethidium bromide.

### Gel extraction

The PCR products obtained were extracted using QIAquick gel extraction kit (Qiagen, Valencia CA, USA). Thirty microliters of the purified DNA of each sample was sequenced (Macrogen, Netherlands).

### Phylogenetic analysis

The 16S rRNA sequences obtained were compared with known 16S rRNA sequences at National Center for Biotechnology Information (NCBI) database using BLASTn (Basic Local Alignment Search Tool) algorithm obtained from; https://blast.ncbi.nlm.nih.gov/Blast.cgi. Identification of the sequences at both the genus and species level was defined as a 16S rRNA sequence similarity of ≥ 99% with that of the prototype strain sequence in GenBank. The sequences together with reference sequences derived from the Genbank were aligned using CLUSTAL W. The topology distance and probability of phylogenetic tree were determined using Mr. Bayes software. The topological robustness of the trees was evaluated by a bootstrap analysis involving 10,000 replications. The tree was then visualized using fig tree software v. 1.3.1.

### *Salmonella* antimicrobial susceptibility tests

Antibiotic susceptibility testing was tested using the Kirby-Bauer disk diffusion method according to the Clinical and Laboratory Standards Institute (CLSI) guidelines. Briefly, pure colonies, bacterial suspension were placed in test tubes and their turbidity adjusted to 0.5McFarland turbidity standards. The diluted bacterial suspensions were then transferred onto Mueller-Hinton agar plates using a sterile cotton swab and seeded uniformly. Antibiotic impregnated discs were then placed to the plate surfaces using sterile forceps. The plates were incubated aerobically at 37 °C for 24 h and susceptible *E. coli* (ATCC 25922) was used as a control. A total of 8 selected antibiotic disks were used which contained the commonly used antibiotics namely; tetracycline (100 μg), nitrofurantoin (200 μg), nalidixic acid (30 μg), streptomycin (25 μg), sulphamethoxazole (200 μg), cotrimaxazole (25 μg), gentamycin (15 μg) and ampicillin (25 μg). Zones of inhibitions were measured to determine whether the bacteria were susceptible, intermediate or resistant in comparison to CLSI critical points.

## Results

### *Salmonella* prevalence and serotypes

**Fig. 1:**
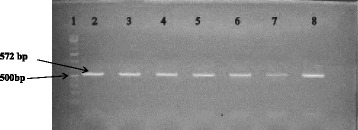
Agarose gel analysis of PCR (572 bp) of *Salmonella* isolates. Lane 1: 100 bp ladder; Lane 2: Positive control; Lanes 3, 4, 5, 8: *S*. Typhimurium; Lane 7: *S*. Enteritidis

Salmonella was isolated in 31 (4%) out of the total 740 samples collected and this comprised of 7 (3.8%) pigs faeces, 4 (2.6%) cattle faeces, 7 (3.6%) chicken cloacal swabs and 13 (5.9%) eggs. The PCR product obtained was 572bp (Fig 1). According to the NCBI blast, 2 serovars of *Salmonella* were identified: 18 out of 21 samples were identified to be *S.* Typhimurium (85.8%) while 3 out of 21 (14.2%) of the samples were identified to be *S.* Enteritidis. All the cattle and chicken positive samples contained *S.* Typhimurium, 25% (¼) of the positive pig samples and 16.7% (1/6) of the positive egg samples contained as *S.* Enteritidis while 75% (¾) as of the pig samples and 83.3% (5/6) of the egg samples were identified to be *S.* Typhimurium.

### Antibiotic resistance profiles of *Salmonella* isolates

**Fig. 2:**
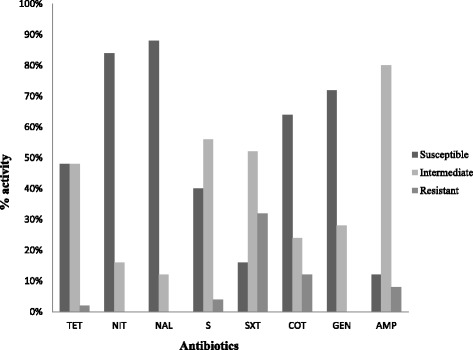
Percentage activity of *Salmonella* isolated from eggs and fecal matter of cows, pigs and chicken to various antibiotics. The activity was grouped as susceptible, intermediate or resistant to the following drugs: tetracycline (TET), nitrofurantoin (NIT), nalidixic acid (NAL), streptomycin (S), sulphamethoxazole (SXT), cotrimoxazole (CoT), gentamycin (GEN) and ampicillin (AMP)

Antimicrobial resistance was identified in 40% of *Salmonella* isolates whereas 20% were resistant to: sulphamethoxazole cotrimoxazole, streptomycin sulphamethoxazole and tetracycline sulphamaethoxazole. In addition, intermediate resistance was observed against nitrofurantoin (84%), ampicillin (76%), sulphamethoxazole (52%), streptomycin (40%), tetracycline (36%), gentamycin (28%), co-trimoxazole (20%) and nalidixic acid (12%) (Fig. 2).

### Phylogenetic analysis

**Fig. 3:**
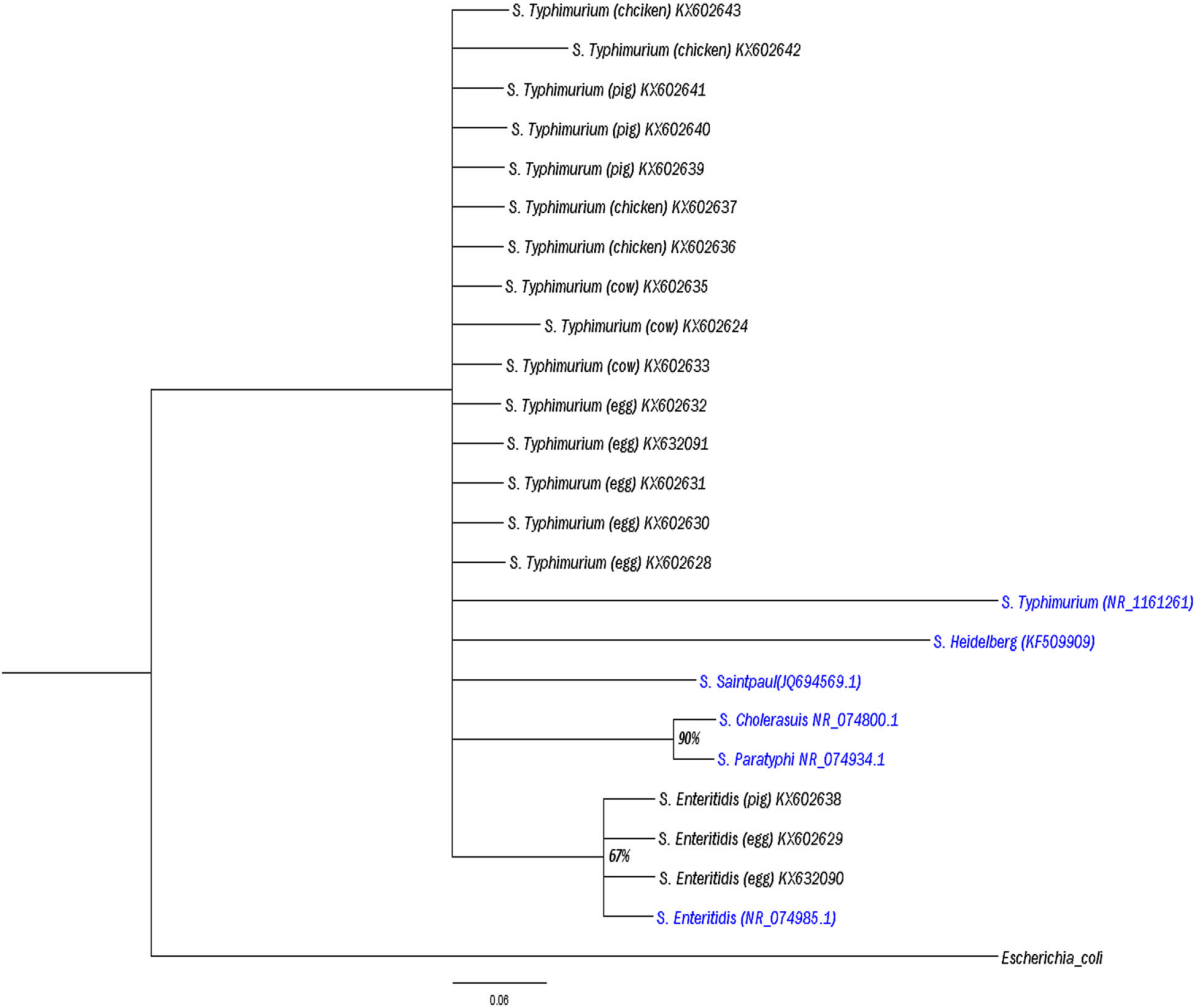
A phylogenetic tree based on 16S rRNA sequences of *Salmonella* isolates. The phylogeny was inferred by Bayesian method using the Markov Chain Monte Carlo (MCMC) method from an alignment performed using Bioedit. The Phylogenetic tree was visualized using Fig Tree v. 1.3.1. Numbers at the nodes show percentage of posterior probabilities indicating topological robustness

*S.* Typhimurium and *S.* Enteritidis were identified and formed two clades in the phylogenetic tree (Fig. 3). *Escherichia coli* was used to be the root the tree where *S.* Enteritidis clade showed a 68% probability from the majority *S.* Typhimurium clade. A 91% probability between the *S.* Choleraesuis and *S.* Paratyphi that were used as reference sequences in this analysis was observed. The branch length it appears that more variation has occurred in *S*. Typhimurium human isolate (*S*. Tm NR074800.1) than the *S.* Typhimurium field samples used in this study.

## Discussion

The study aimed to determine the prevalence of *Salmonella*, the genetic relatedness of the serovars as well as determine their antibiotic resistance patterns. The study findings show that *Salmonella* was detected in the faecal matter of seemingly healthy animals and animal products meant for human consumption as well as in animal products. Before the animals are slaughtered a certified veterinarian inspects the health status of the animal by checking the skin fur, faecal matter, physical appearance and other clinical parameters. The prevalence of *Salmonella* was pigs (3.8%), cows (2.6%), chicken (3.6%) and eggs (5.9%) that are meant for human consumption. Out of the positive samples isolated, two serovars were detected: *S*. Enteritidis and *S.* Typhimurium. Resistance was detected in 40% of the samples.

The prevalence of *Salmonella* in eggs (5.9%) was higher in this study than in the Ethiopia study which established a prevalence of 4.69% [18]. The presence of *Salmonella* in eggs in Kenya therefore is a concern because several outbreaks have been attributed to consumption of contaminated eggs in other parts of the world [20]. Most food-borne *S.* Enteritidis infections are associated with the consumption of raw eggs and foods containing raw eggs such as homemade ice cream, mayonnaise and others egg products [18, 21]. There are two methods of contamination: on the outer shell and internally. Internal contamination can be as a result of the contamination through the eggshell or direct contamination of egg contents before oviposition [22]. The detection of *Salmonella* in eggs demonstrates that improvements need to be made in controlling *Salmonella* transmission in farms.

The prevalence of *Salmonella* in chicken in this study was 3.6%. *Salmonella* contamination rates for chicken reported in literature vary from 0.8 to 11% in Ethiopia [17, 23, 24] and Nigeria [25, 26]. The results of this study are comparable to results obtained in Tanzania [27]. The lower prevalence of 0.8% in Aragaw et al. [23] could be due to the fact that pre enrichment was not done. Pre enrichment helps to proliferate or regenerate cells thus increasing their viability when cultured on a solid medium [28]. The differences in prevalence could also be due to the geographical region, the type of chicken screened whether local indigenous or the exotic breeds. This study corroborates the work done in an Ethiopian study [17] where there was a higher prevalence of *Salmonella* in the indigenous chicken 71.4% compared to the grade chicken 28.6%. The levels of *Salmonella* in poultry can vary depending on the method of isolation, country, the nature of the production system and the specific control measures in place [12].

The prevalence of *Salmonella* in cows was lower in this study compared to studies done in Ethiopia [19]. This could be due to differences in environment, geographical distribution as well as husbandry practices. In the above studies a higher prevalence has been observed amongst dairy cattle compared to beef cattle [19].

The levels of Salmonella found in pigs in this study (3.8%) is comparable to those obtained by in Korea [29] but is much lower than previously reported in Kenya [16] and Burkina Faso [12]. The disparity in the prevalence for the Kenyan study could be due to better husbandry practices that have caused a reduction in the Salmonella prevalence in the pigs.

In this current study, two [2] serovars were identified: *S*. Typhimurium and *S*. Enteritidis. These two serovars are most commonly associated with food products and are the major causes of Salmonellosis in humans worldwide [30, 31]. The serovars identified in this study are contrary to the study done in Kenya where *S.* Heidelberg, *S*. Agona and *S.* Saintpaul were the most common isolated serovars in pigs [16]. In cows *S.* Typhimurium and Newport were the most isolated in Ethiopia [32] while in another Ethiopian study *S*. Anatum and *S.* Newport were the most commonly isolated [33]. In eggs *S.* Enteritidis was the isolated serovar in Ethiopia whereas in Australia *S.* Typhimurium is the most isolated serovar [30]. These results highlight the complexity of the global epidemiology of *Salmonella* as the frequency and occurrence of different serovars changes over time in countries and regions. Shifts in prevalence may follow introduction of the strain through animal feed and livestock trade [34].

Genotypic identification methods are emerging as an alternative or complement to established phenotypic identification procedures. For bacteria, 16S rRNA gene sequence analysis is a widely accepted tool for molecular identification [35]. From the phylogenetic tree (Fig. 2) the two serovars: *S*. Typhimurium and *S.* Enteritidis formed two distinct clades. From the branch length it appears that more variation has occurred in *S*. Typhimurium human isolate than the *S.* Typhimurium field samples used in this study.

Antibiotic resistance is the evolutionary response by bacteria to the strong selective pressure that results from exposure to antibiotics [36]. The *Salmonella* isolates in this study were susceptible to most of the easily accessible and cheaper drugs such as tetracycline while resistance was observed against sulphamethoxazole and cotrimoxazole. This could be an indicator of the acquisition of the resistance genes for those drugs due to the indiscriminate use of these 2 drugs at recommended doses or at sub therapeutic doses in feed additives to promote growth creating on farm selection of antimicrobial resistant strains [6]. Two of the isolates were resistant to sulphamethoxazole and not to cotrimoxazole which is a combination of sulphamethoxazole and trimethoprim (a folic acid analogue). Cotrimoxazole works by inhibiting 2 steps in the enzymatic pathway for bacterial folate synthesis. The isolates therefore seem to have not acquired the trimethoprim resistance, *dhfr* genes that encode altered dihydrofolate reductases that reduced affinity for the antimicrobial agent, allowing folic acid biosynthesis to occur in the presence of trimethoprim [37]. There was also a high percentage of isolates that were intermediately resistant to the panel of antibiotics tested. As compared to a previous study done by Kariuki et al. [21] where all the isolates from animals were susceptible to the commonly used drugs, our study findings could be indicative of increasing resistance towards the commonly used drugs and a cause of concern in the treatment of NTS. The detection of resistance in the samples in this study shows that there could be an indicator of the increased use of the antibiotics at sub-therapeutic levels or prophylactic doses which may promote on-farm selection of antimicrobial resistant strains.

## Conclusion

The study showed that animal and animal products carry *Salmonella*. Isolation of *Salmonella* Typhimurium and *Salmonella* Enteritidis that exhibit resistance or intermediate sensitivity to commonly used antibiotics from seemingly healthy animals and eggs poses a significant public health threat because it is indicative that there is presence of zoonotic organisms that have the potential of entering the food-chain especially in Kenya. The emergence of antimicrobial resistant *Salmonella* strains is a problem and prudent use of antibiotics in animal husbandry and human therapy should be encouraged to help conserve the limited options of antibiotics available. Continual monitoring and surveillance to detect drug resistant *Salmonella* strains should be done and this should guide the administration of effective antibiotics accordingly.

## Abbreviations

AMP: Ampicillin
CoT: Cotrimoxazole
GEN: Gentamycin
NAL: Nalidixic acid
NIT: Nitrofurantoin
NTS: Non typhoidal *Salmonella*
S: Streptomycin
SXT: Sulphamethoxazole
TET: Tetracycline
